# Flexible pressure and temperature dual-mode sensor based on buckling carbon nanofibers for respiration pattern recognition

**DOI:** 10.1038/s41598-022-21572-y

**Published:** 2022-10-19

**Authors:** Zhoujun Pang, Yu Zhao, Ningqi Luo, Dihu Chen, Min Chen

**Affiliations:** 1 School of Physics, Sun Yat-Sen University, Guangzhou, 510275 People’s Republic of China; 2 State Key Laboratory of Optoelectronic Materials and Technologies, School of Electronics and Information Technology, Sun Yat-Sen University, Guangzhou, 510275 People’s Republic of China; 3 School of Materials and Energy, Guangdong University of Technology, Guangzhou, 510006 People’s Republic of China

**Keywords:** Health care, Materials science

## Abstract

Breathing condition is an essential physiological indicator closely related to human health. Wearable flexible breath sensors for respiration pattern recognition have attracted much attention as they can provide physiological signal details for personal medical diagnosis, health monitoring, etc. However, present smart mask based on flexible breath sensors using single-mode detection can only detect a relatively small number of respiration patterns, especially lacking the ability to accurately distinguish mouth breath from nasal one. Herein, a smart face mask incorporated with a dual-sensing mode breathing sensor that can recognize up to eight human respiration patterns is fabricated. The breathing sensor uses novel three dimensional (3D) buckling carbon nanofiber mats as active materials to realize the function of pressure and temperature sensing simultaneously. The pressure model of the sensors shows a high sensitivity that are able to precisely detect pressure generated by respiratory airflow, while the temperature model can realize non-contact temperature variation caused by breath. Benefit from the capacity of real-time recognition and accurate distinguishing between mouth breath and nasal breath, the face mask is further developed to monitor the development of mouth breathing syndrome. The dual-sensing mode sensor has great potential applications in health monitoring.

## Introduction

A great effort has been taken to mitigate the rapid worldwide spread of the Coronavirus disease 2019 (COVID-19) but the vaccine is insufficient to curb the spread of the novel coronavirus that mutates rapidly^1,2^. Wearing mask in public places has been recommended by the World Health Organization and widely imposed by the majority of countries to prevent the spread of the disease and protect the health of individuals in the COVID-19 pandemic. However, wearing masks for a long time may cause possible adverse effects. For asthmatic patients or children, breathing difficulty or acute respiratory disease would be imperceptible. Severe breathing problems could cause abnormal mouth breathing and even cause respiratory failure, which is necessary to alarm in time. Therefore, daily breath monitoring based on portable wearable devices is of great significance in providing early warning of abnormal breathing condition for children or patients with respiratory problems. Respiration is an essential physiological indicator that plays an important role in clinically evaluation of individual’ health performances^3,4^. Usually, when nasal breathing is difficult, people tend to breathe through their mouths to increase the intake of air. The children with asthma may be more likely to breathe through their mouths^5^. The tendency of habitual or long-term mouth breathing not only adversely affects the child's jaw development, skull shape and tooth occlusion, but also associates with sleep apnea syndrome. Real-time monitoring of breathing is necessary for early multidisciplinary diagnosis of this population to prevent the development of mouth breathing syndrome^5,6^. An increased breathing resistance caused by a mask can exacerbate this problem. Moreover, there are many results suggest a significant association between mouth breathing and asthma^7^. Thus, continuous monitoring of breathing conditions of users in daily life, especially with accurate recognition of nose and mouth breathing, can provide an opportunity for personal healthcare monitoring, early warning of acute respiratory disease and medical diagnosis, etc.

A smart mask based on flexible breath sensor is an important way to realize continuous breath monitoring and pandemic prevention. At present, many studies have proposed flexible sensor based on different sensing mechanism to realize breath monitoring, such as humidity, pressure or temperature sensors^8–13^. Very recently, Someya et al. has designed a smart face mask that integrates the ultrathin and lightest electrostatic pressure sensor to realize breath-monitoring^13^. Dao et al. have demonstrated a wearable thermal flow sensor for real-time human respiration by using flexible CNT yarns as hotwires^8^. Peng et al.have reported a self-powered electronic skin (e-skin) based on a triboelectric nanogenerator for real-time respiratory monitoring and obstructive sleep apnea–hypopnea syndrome diagnosis^14^. There are also many humidity sensors have been fabricated to monitoring respiration by detecting the variation of the amount of water in the inhaled and exhaled gases^3,10,15–18^. However, these breath sensors based on single-mode detection can only monitor a relatively small number of breathing patterns, especially lack of the capacity of distinguishing mouth breath from nasal one. Their unitary detecting functionality cannot satisfy the increasing demands for various breath pattern monitoring. There are limitations associated with single-mode detection sensor due to interference between mouth and nose breathing. For example, when the flow intensities of nasal deep breathing and mouth breathing are at the same range, it is difficult to distinguish since they are under the same frequency. Although the detection of different breathing status in a single-mode sensor unit can be realized roughly, signal coupling and mutual interference reduces measurement accuracy and requires calibration when working conditions change^11,19^. In addition, these existing dual-mode sensors are not sensitive enough to simultaneously monitor different physical stimuli caused by breathing airflow^19–24^. It is desirable for a kind of sensing material with multi-sensing capability to simultaneously monitor multiple vital signs of human body via constructing different sensing model structures^25,26^.

In this work, we proposed a smart face mask incorporating a double module detection of pressure and temperature for monitoring breath information, which achieves accurate discrimination between mouth and nasal breathing. By constructing different sensing model structures, a novel flexible carbon nanofibers mats with superior mechanical properties and temperature sensing performance are used as the active materials for both the pressure and temperature sensing module. The pressure sensing module demonstrate a high sensitivity that are suitable for detecting pressure generated by respiratory airflow and can detect various physiological signals of the human body. The temperature sensors can realize non-contact temperature detection by sensing small temperature changes caused by mouth and nose breathing. We demonstrated that the smart face mask can continuously monitor and analyze breath conditions including eight breath-patterns, such as nasal normal breath, nasal shallow breath, nasal deep breath, nasal fast breath, cough, breath-holding, mouth normal breath and mouth slow breath. Compared to previously reported breath-monitoring techniques, the proposed smart face mask offers two advantages: (1) It achieves accurate discrimination between oral and nasal breathing; (2) The dual-mode sensing enables an unprecedented variety of respiration patterns monitoring, allowing a more detailed analysis of the human body's physiology. The smart face mask has great potential in applications of early warnings or diagnoses of breathing-related diseases and is beneficial for maintaining personal health.

## Results and discussion

**Figure1:**
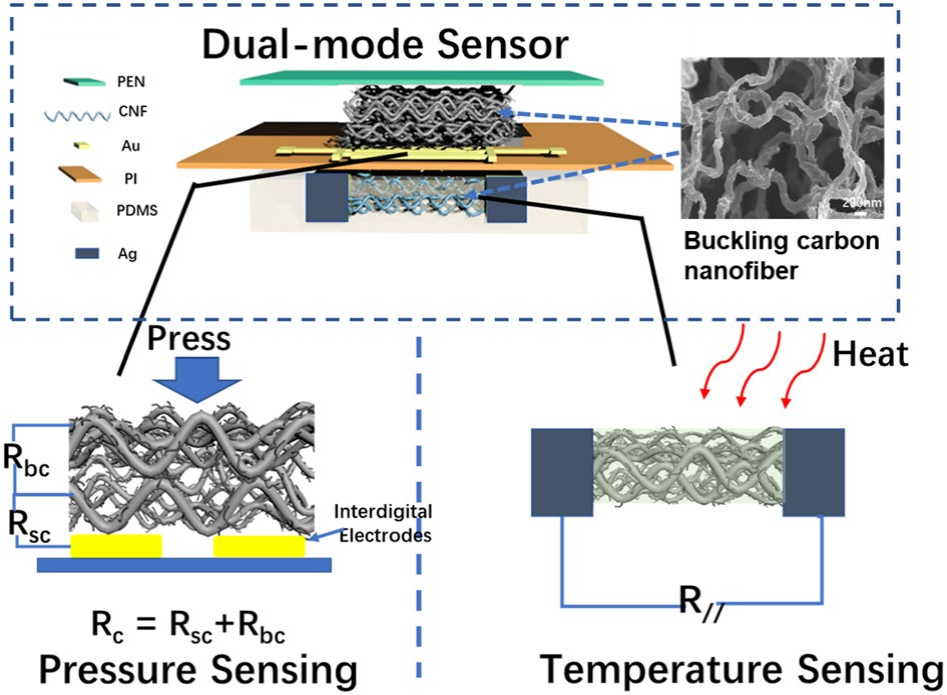
Schema of the structure of a combo dual-mode sensor assembled on a mask to detect pressure and temperature changes caused by breathing.

As shown in Fig. 1, by constructing different sensing model structures, a CNF mats composed of thousands of carbon nanofibers is used as the active materials for the combo sensors to realize simultaneous sensing of pressure and temperature. The novel carbon nanofibers with buckling nanostructure has a large three-dimensional interspace and high specific surface area of (244m^2^/g) (Fig. S1a, Supplementary Information). These nanofibers are decorated with projecting tentaclelike carbon nanotubes possess (Fig. S1b, Supplementary Information), which would be sensitive to different physical stimuli. The CNF mats are achieved by rapid annealing of a hybrid polyacrylonitrile electrospinning nanofibers. The preparation process of dual-mode sensor is shown in Fig. S2, Supplementary Information.

In the pressure model, the CNF mats was placed on the top of interdigital electrodes. For the piezoresistive sensor, the total resistance includes the bulk resistance (*R*_b_) of the conductive composite and contact resistance (*R*_c_). Based on tunneling effect and contact mechanic, it is generally believed that the *R*_c_ is much greater than *R*_b_, thus, the bulk resistance can be neglected ^27^. Thus, the total resistance is dominated by the *R*_c_, including the contact resistance between the CNF defined as bulk contact resistance, *R*_bc_, as well as between the CNF and electrodes defined as surface contact resistance, *R*_sc_. The total transverse contact resistance is

1 $$R_{c} = R_{bc} + R_{sc}$$

The point contact channel formed can be described as analogous to an electron tunneling current when pressure is applied. According to the electrical contact fundamentals, the increased real contact area generated merely by elastic deformation can be described with a loaded bearing area as $A_{b} = \frac{F}{H}$^28,29^, where F is the applied force, H is the Meyer hardness of the softer material^30^. The real contact area defined as A_c_ = A_b_ + A_0_.

The contact resistance can be described as:

2 $$R_{c} = \frac{{\rho_{1} + \rho_{2} }}{4}\sqrt {\frac{\pi }{{A_{0} + \frac{F}{H}}}}$$

where *ρ*_*1*_ and *ρ*_*2*_ are the resistivity of the two contacting materials, A_0_ is the contact area between two contacting material under zero loading force. According to the definition of pressure sensor sensitivity:

3 $$S = \frac{{\Delta I/I_{0} }}{\Delta P} = \frac{{\delta \left( {\left( {\frac{1}{{R_{c} }} - \frac{1}{{R_{c0} }}} \right)/\frac{1}{{R_{c0} }}} \right)}}{\Delta P} = \frac{{\sqrt {1 + \frac{{\Delta A_{b} }}{{A_{0} }}} - 1}}{\Delta P} \approx \frac{{\sqrt {\frac{{\Delta A_{b} }}{{A_{0} }}} }}{\Delta P}$$

The results of Eq. (3) theoretically demonstrates that pressure sensitivity of the pressure model is independent of the resistivity of materials, which thus has very little interference from temperature. In this model, as the buckling carbon nanofibers forms a large three-dimensional space and has a large adjustable contact area, the initial contact area *A*_0_ is much smaller than *A*_b_. Therefore, the piezoresistive sensitivity is determined by the change ratio of loading bearing area to unit pressure. The piezoresistive behavior of the sensor originates from the variation of the contact areas or points between the CNF as well as between the CNF and electrodes. What is more, there are numerous sensitive points formed by the projecting carbon nanotubes on the carbon fibers. Once the buckling nanofiber mats receives a transverse pressure, these fibers and electrodes contact each other to form high conductive path, resulting a large change of Rcb and Rcs, Therefore, a high sensitivity can be achieved in this piezoresistive sensing mode.

In temperature sensing module, the two ends of parallel carbon nanotubes are coated with conductive silver paste as electrodes, and then the whole CNF mat was coated by PDMS serve as the encapsulation layer. The CNF are wrapped by the PDMS and form a transverse dielectric insulating layer to suppress the mutual interference caused by pressure stimulation. The equivalent circuit of a single temperature sensor and measurement methods are shown in Fig. 1. According to the formula, the monoblock CNF mats is regarded as a resistance, and the longitudinal resistance R_//_ can be expressed as

4 $$R_{// } = {\uprho }\frac{L}{{S_{// } }} = {\uprho }\frac{L}{{n S_{cnf} }}$$

where S_//_ is the total cross section monoblock CNF in horizontal direction, and the number of CNF is n, and cross section of each fiber is *S*_cnf_. In this temperature sensing structure model, due to the inherent hardness of the carbon material, the change in the cross section of each carbon nanofiber is negligible under applied pressure. Therefore, the resistance R_//_ are mainly decided by temperature-induced change in resistivity ρ. Furthermore, the PDMS coating on CNF as a transverse dielectric insulating layer avoid pressure interference, so the contact resistance is negligible in temperature sensing model.

**Figure2:**
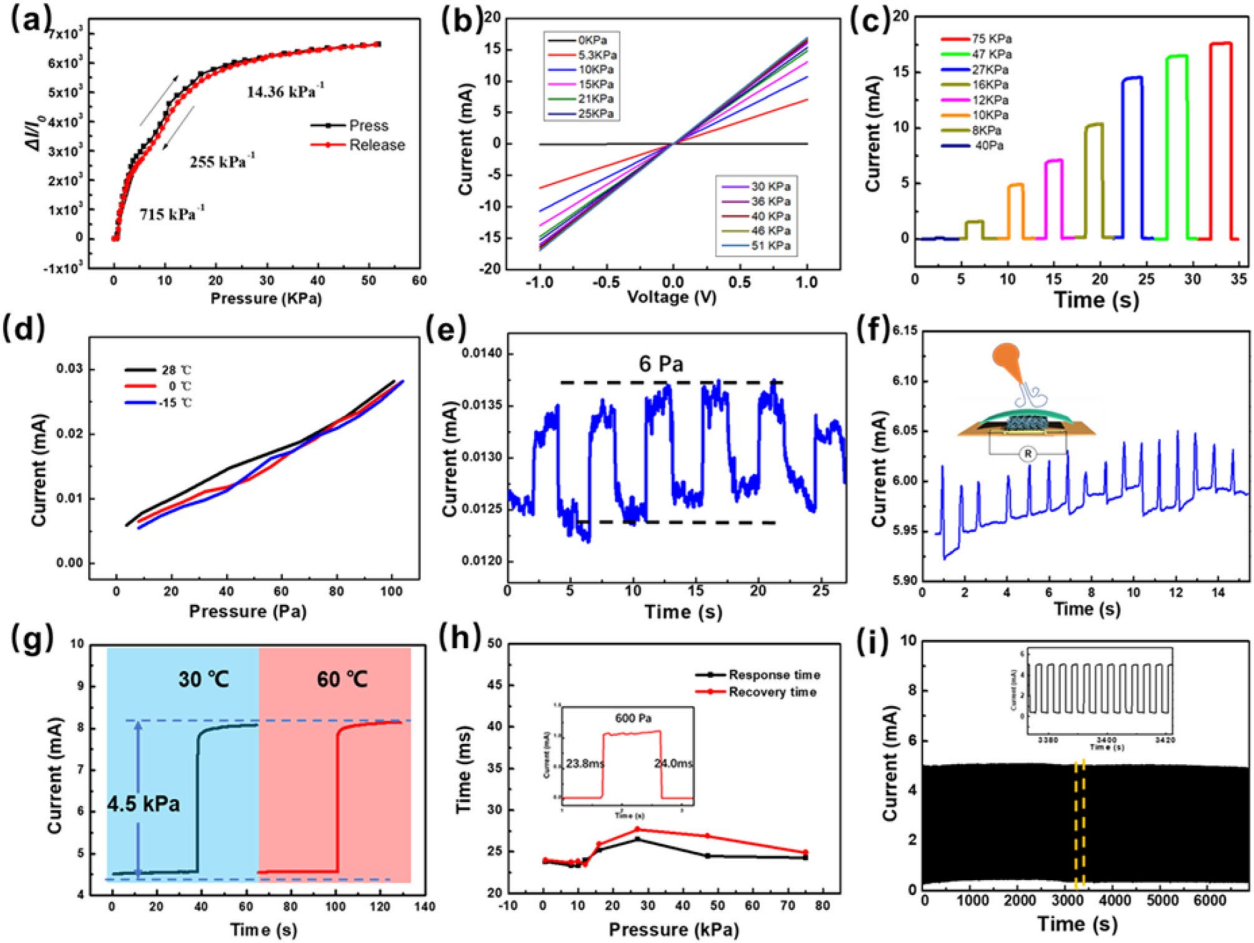
(**a**) Relative current change ratios as a function of the pressure. (**b**) Current–voltage curves under different pressure. (**c**) Responding current under different pressure. (**d**) Current curve under low pressure range. (**e**) Current response to repeat low pressure. (**f**) Open-circuit voltage versus time under the stimuli of an airflow. (**g**) Current response under different temperature (**h**) Response speed of pressure sensor. (**i**) Current response to continuous pressure train.

To accurately measure the breath conditions, the flexible breath monitoring sensors should be stable and highly sensitive to respiratory airflow at low pressure. Figures 2a shows the sensitivity curve of the pressure sensor in a pressure loading–unloading test cycle. The pressure sensitivity is defined as S = (ΔI/I_0_)/ΔP, where ΔI denotes the relative current change, I_0_ is the initial current, and ΔP is the difference in pressure load. Before applying pressure, the contact area between the carbon nanofibers or between carbon nanofibers and electrode is very small, corresponding to a high resistance state. The sensitivity curve reveals a highest sensitivity value (715 kPa^−1^) in the low-pressure range (0–5 kPa) for the numerous carbon fibers contact with each other to create highly conductive path. In the subsequent increasing pressure range (5–20 kPa), with the increasing of contact area between the carbon nanofibers, the sensor exhibited a sensitivity of 255 kPa^−1^. In the high-pressure regime (> 20 kPa), the fibers are pressed together closely and the sensor exhibited a relatively low sensitivity of about 14.36 kPa^−1^. A sensor device without CNT exhibits a pressure sensitivity four orders of magnitude lower than that with CNT (Fig. S3, Supplementary Information), indicating that the decoration of carbon nanofibers with CNT is crucial for the sensing performance. Figure 2b shows the current–voltage curves of the pressure sensor for different pressures, with voltages ranging from −1 to 1 V. The observed curves are consistent with Ohm’s law. We tested the repeated current-response for different pressures (Fig. 2c) and found an excellent steady sensing-performance and repeatability for the sensors. A pressure sensor that is easily triggered by low pressure is desirable for flow pressure detection of smart masks. The real time current-pressure curve in Fig. 2d shows a good linearity in low pressure range. Figure 2e shows the detection of extremely small pressure variation of about 6 Pa in the background pressure of about 30–40 Pa. As indicated in Fig. 2f, driven by airflow pulse with a pressure of ~ 120 Pa, the pressure sensor was able to generate a periodic current peak. To further demonstrate the merit of ultrahigh sensitivity, a flexible pressure sensor is attached to the skin above throat to recognize words with different numbers of syllables (Fig. S4, Supplementary Information).

A test applying the same pressure at different temperatures is carried out to demonstrate the capacity of suppressing interference from temperature variation in the pressure detecting mode. Figure 2g shows that pressure responses under an increase of 4.5 kPa at 30 °C and 60 °C are extremely similar. Also, a slight pressure of 10 Pa applied to the pressure sensor results in an almost unchanged current increment △I as temperature varies from 25 to 60 °C (Fig. S5, Supplementary Information). Therefore, the measurement of pressure, which is only related to current increment △I, is hardly influenced by the variation of temperature. The dynamic response speed is another important parameter of breath sensor. The response- and recovery-times are calculated in Fig. 2h and the several detailed response curves under different pressures are shown in Fig. S6 (Supplementary Information). The response time under different pressure is in the range of 20–30 ms, which is fast enough for the breath detection. The cycle test of pressure sensor shown in Fig. 2i demonstrates excellent operating stability and durability under 2000 continuous loading/unloading train, since almost no degradation is found during the test period. In addition, to demonstrate the practical application of the sensor with respect to detecting physiological signals in humans. The sensor is attached to the wrist to monitor the arterial pulse signal, which clearly display three typical main waves of the P-wave, T-wave, and D-wave (Fig. S7, Supplementary Information).

**Figure 3:**
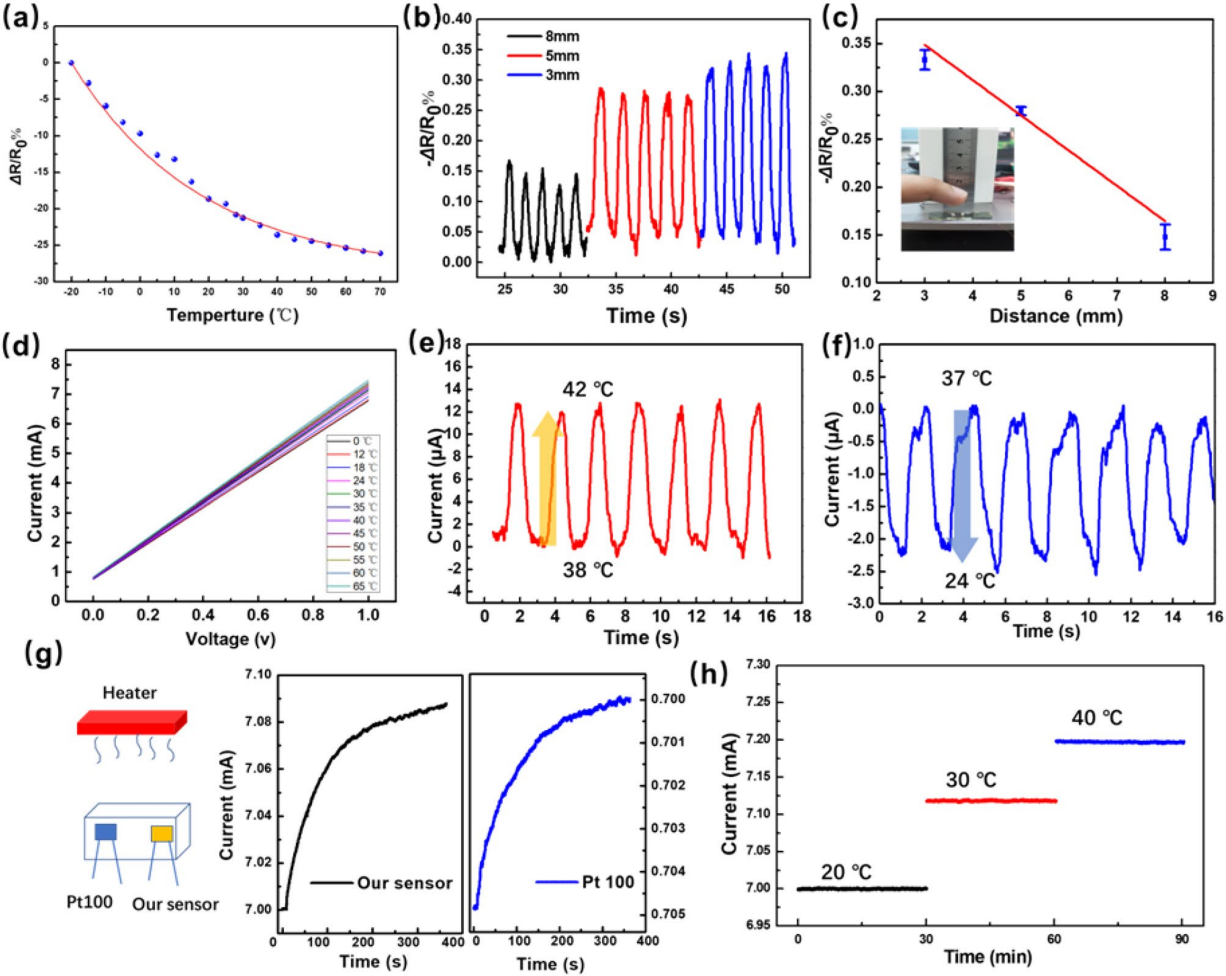
Temperature-sensing properties of the double module sensor. (**a**) The linear correspondence of the absolute value of electric resistance variation rate with temperature from 4 to 70 °C. (**b** and **c**) Influence of minimum distance between the finger surface and sensor on the relative resistance change. (**d**) I-V Curves denote the current of the sensors in response to temperature variated from 0 to 65 °C. Temperature-discrimination ability of the sensor during repeatedly approaching and moving away from the (**e**) heat source object, and (**f**) cool source object. (**g**) Temperature response curves recorded by our sensor and the PT100 temperature sensor. (**h**) Temperature–time curve at 20 °C, 30 °C and 40 °C.

Despite the superior piezoresistive performance demonstrated in the piezoresistive model, the temperature-sensing properties are also an important model in combo sensor for the accurate detection of human breathing signals. Recently, the good electrical response to the temperature variations of CNT has been reported and CNT nanocomposites with polymers and other materials applied in temperature sensor have attracted considerable attention^31–33^. In our work, electrospinning carbon fibers are modified with tentacle-like carbon nanotubes and wrapped in PDMS. The modified antenna of carbon nanotubes on the 3D carbon fiber can receive thermal radiation and cause the change of electrical resistivity. As discussed in the temperature-sensing model above, the resistance R_//_ is mainly decided by temperature-induced change in resistivity ρ. Therefore, the nonlinear temperature dependency of resistances can be described by the following growth exponential^33^:

5 $$R_{// } = R_{0} {\text{exp}}\left( {\frac{{E_{a} }}{2KT}} \right) = R_{0} {\text{exp}}\left( \frac{B}{T} \right)$$

where *E*_a_ is the thermal activation energy, K is the Boltzmann constant, and B is the thermal index. The relative change of resistance with temperature is plotted in Fig. 3a, which shows increase in resistance with temperature and the highest sensitivity of 0.22%/°C. Figure 3d reveals the change of slope of *I-V* curves (1/R) of the sensor when temperature raise from 0 to 65 °C, and shows the linear correlation within the range of 0–1 V of the temperature sensor. For breathing sensing, the sensor is required to have high sensitivity and non-contact detection function. Firstly, the sensitivity of the contactless sensor is examined by changing the distance between the finger and the sensor to produce a small change in temperature. As show in Fig. 3b and c, smaller distance means higher relative temperature, so the relative resistance change increases stepwise as the finger’ distance decreases from 8 to 3 mm. In addition, the Fig. 3e and f shows the relative current change as the sensor approaching/retreating from the heat source (from 38 to 42 °C) and cool source (from 37 to 24 °C). As a comparison, a widely used commercial PT100 temperature sensor and our temperature sensors were placed in still air environment and at a certain distance from the heater to record the temperature change in real time (Fig. 3g). As shown in Fig. 3g, the temperature curve from our sensor was totally identical to that from the PT100 temperature sensor during the non-contact heat transfer process. Furthermore, to verify the running stability of temperature sensing mode, the current monitoring of the sensor was carried out at different temperatures for a long time. As shown in Fig. 3h, the temperature sensor works very smoothly at 20 °C, 30 °C and 40 °C, respectively. To further demonstrate the application of a wearable flexible thermometer, the sensor was installed on the subject’s forehead to quantitatively reveal human skin temperature variation realized by the heat source approaching/retreating from the subject’s forehead (Fig. S8, Supplementary Information).

**Figure 4:**
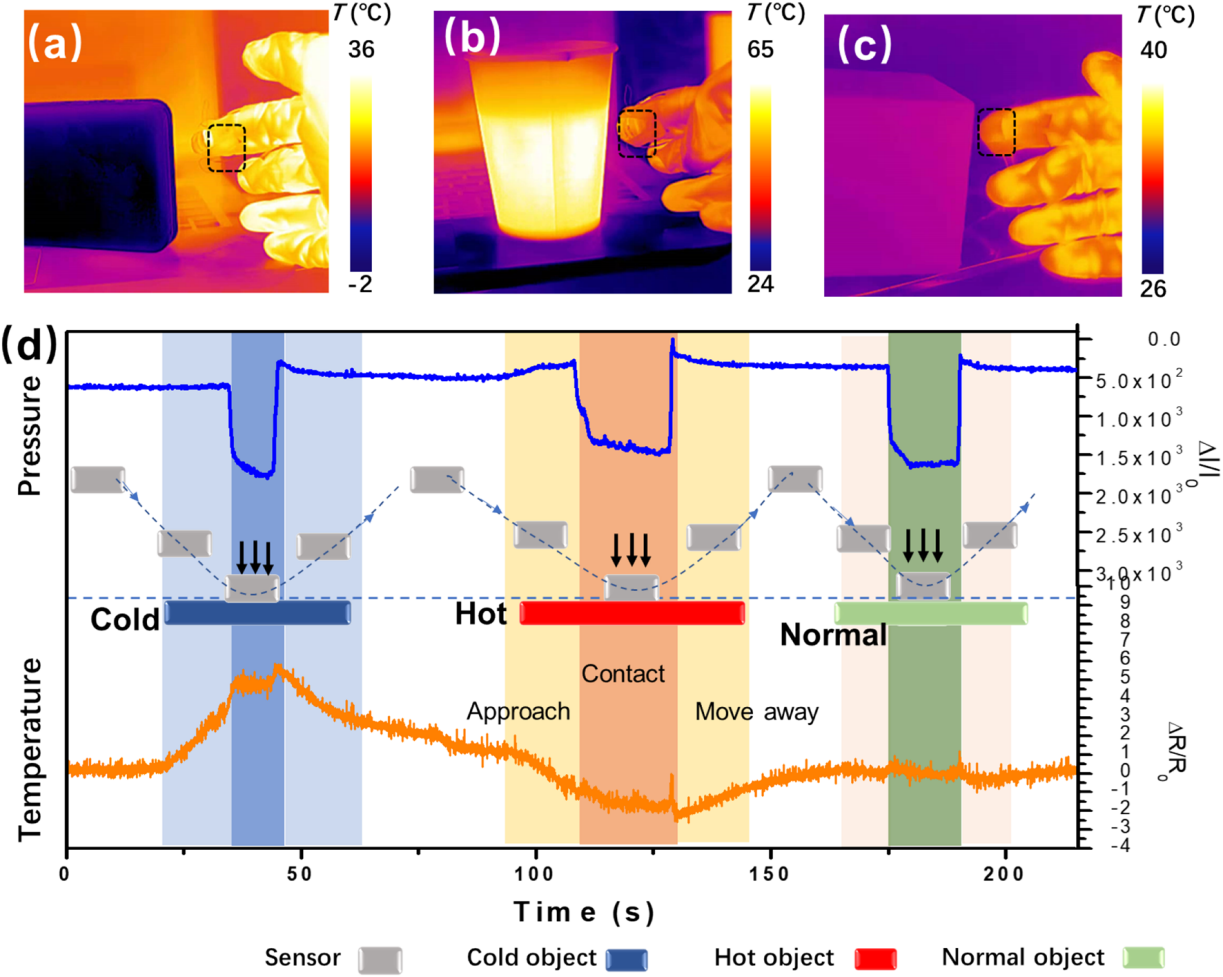
Infrared thermal images of sensor attached on glove approaching (**a**) cold object, (**b**) hot object and (**c**) normal object. (**d**) Plots of the real-time output current and resistance responses of the sensor.

The noncontact temperature detection capability is indispensable for a flexible sensor assembled in smart mask for breath monitoring. To demonstrate that the two sensing modes of the combo sensor can work independently without mutual interfering, a test to monitor both temperature and pressure is proposed. Figure 4a–c, show the infrared thermal images of dual-mode sensor attached on a glove approaching or contacting the cold object, hot object and normal object, respectively. Considering the thermal diffusion in air, contact-free sensing of temperature can be obtained when the temperature difference exists between the object and the sensor. As shown in Fig. 4d and Movie S1 (Supplementary Information), when the sensor approach hot or cold objects, an obvious increase/decrease in temperature was detected after the distance decreased to 1 cm. But the sensor demonstrates a negligible temperature vibration when approaching the normal object at room temperature. When mechanical contact occurred between sensor and object, the monitored pressure increased rapidly from 0.8 to 2 kPa and the surface temperature maintained at a stable value (results of another similar experiment can also be seen in Figure S9), confirming again the very little interference from pressure to the temperature sensor. In addition, the repeated test of detecting temperature and pressure stimuli of objects at different temperatures shows in Fig. S10 (Supplementary Information), which the reveal a stability of dual-mode sensing. The above results clearly prove that the individual sensing modes of the dual-mode sensor can operate independently without mutual interference. This unique function of our dual-mode sensor enables promising applications in smart mask for breath monitoring and artificial intelligent electronic skin^34^.

**Figure 5:**
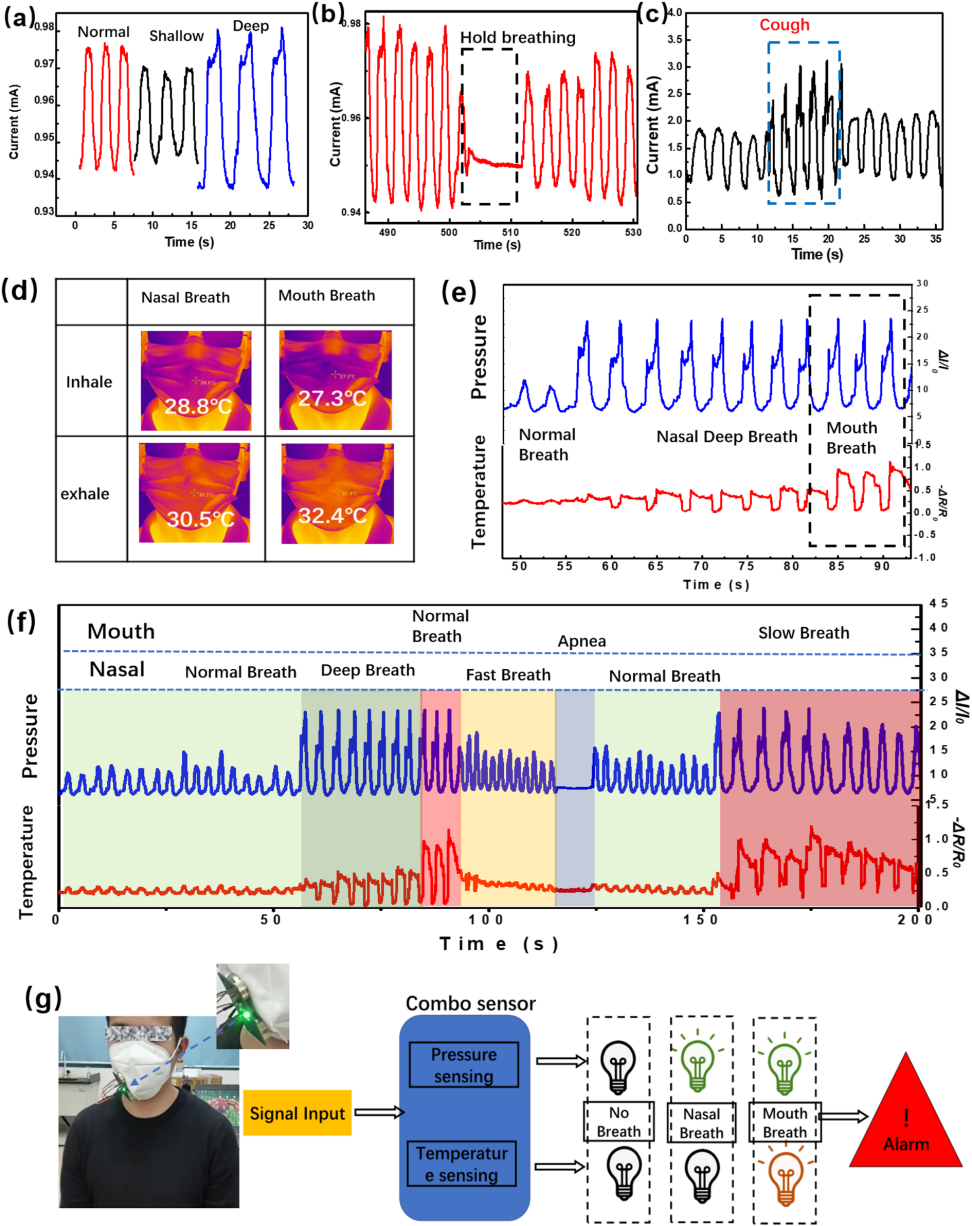
(**a**) Detection signals for breathing pattern with different pressure intensities, (**b**) hold the breath, and (**c**) coughing. (**d**) Infrared images of oral and nasal respiration. (**e**) Pressure and temperature response under nose breathing and mouth breathing conditions. (**f**) Breathing patterns recognized by the response signals to the successive nose and mouth breathing. (**g**) Smart masks distinguish between mouth and nose breathing through the monitoring of temperature.

A smart face mask is fabricated by incorporating a double-model combo sensor. A small measuring circuit with indicator for the real-time display of different breathing states is also integrated. Firstly, for pressure model, different respiration states can be recognized by studying the signals of sensors in response to the pressure variation on the mask caused by the changing airflow during nose breathing. The real-time monitoring data for human nose breathing are shown in the Fig. 5a and Fig. S11 (Supplementary Information). The sensor shows a series of significant peaks, which can be classified as different breathing states (normal breathing, shallow breathing, and deep breath). Each breathing state, which is characterized by different cycle time and the respective peak intensity, can be measured accurately. The respiration states for normal breathing, deep breathing, and shallow breathing have different breathing rates of 18 s^−1^, 12 s^−1^, and 30 s^−1^, respectively. Furthermore, abnormal state of human respiration, such as the apnea and cough, can be accurately measured, as shown in Fig. 5b and c. The pressure sensor was sealed by PEN film and acrylic PSA adhesive, which can prevent the major contact between humidity and active materials of sensor. Even if the sensor was not fully sealed, the hydrophobic surface of the exposed CNF can still effectively resist the permeating of the humidity. As shown in Figure S12, the carbon nanofibers is hydrophobic with contact angle of 130°. For the temperature sensor, the whole CNF was coated by compact PDMS, which forms a physical isolation from the moisture. All in all, the humidity between body and masks has little influence on the performance of dual-mode sensor. Also, the face mask can stably response to breathing pressure in a long period (Fig. S13, Supplementary Information), indicating the reliability of the mask.

The existing smart masks to monitor breathing are mostly based on single-mode flexible sensors. The single mode is prone to errors in monitoring complex breathing, and it is difficult to accurately distinguish mouth breath and nose breath. For example, as shown in the pressure test section in Fig. 5e, it is difficult to distinguish the nasal deep breathing and mouth breathing when the pressure intensity of respiratory airflow is almost uniform. During the COVID-19 pandemic, the double-model breath monitoring with accurate recognition of nose and mouth breathing can provide early warning of dyspnea in asthmatic patients and children. What is more, long-term monitoring of respiration is necessary for early multidisciplinary diagnosis of the children with asthma to prevent the development of mouth breathing syndrome. Therefore, a smart mask that can accurately distinguish nose breathing and mouth breathing have a great potential in practical applications.

The clear recognition of the different breathing state is possible by the accurate measurement of the temperature of the exhaled air and the difference of air pressure simultaneously. The infrared images show the temperature difference produced by mouth breathing and nasal breathing while wearing a mask (Fig. 5d). Nasal breath demonstrates a change of temperature from 28.8 to 30.5 °C during a period of breath, which is smaller than that (27.3–32.4 °C) of mouth breath. The nasal breath and mouth breath can be distinguished by the different variation of temperature in the single process of inhaling and exhaling. This special feature is important when the respiratory airflow pressure intensity of nasal deep breathing and mouth breathing was basically the same. By the complementary monitoring of pressure and temperature, the status of normal nasal breath, nasal deep breath and mouth breath can be recognized, as shown in Fig. 5e. Figure 5f shows the real-time response of double-model sensor in smart mask for human breath detection. Accurate monitor of various breathing patterns, including nasal normal breath, nasal deep breath, nasal fast breath, cough, apnea, and mouth normal breath, mouth slow breath, can be achieved successfully. Combined with the previous breathing patterns monitored in pressure mode, a total of eight breathing patterns can be detected by the dual-mode sensor. The powerful detecting capacity of the double-model sensors allows more detailed analysis of the human body's physiology, which is highly demanded in medical flexible devices.

To demonstrate the practical value of dual-mode sensors, the smart mask has further developed the capacity to display mouth and nasal breathing status in real time, as shown in Fig. 5g and Movie S2, Supplementary Information. This smart face mask is combined with a dual-mode breathing sensor, a power supply unit, a measuring circuit and two indicator light, as shown in Fig. S14, Supplementary Information. The indicator light is off when there is no breathing, while the green indicator lights up when the nose breathes. When mouth breathing occurs, the red and green indicators light is turn on simultaneously. The smart masks allow real-time observation of mouth breathing, thereby very helpful in preventing breathing problems. Moreover, timely correction of abnormal mouth breathing for children can prevent the development of mouth breathing syndrome. It is expected that this breath sensor could be further combined with wireless readout circuit and a mobile application to enable wireless breath-monitoring.

## Conclusion

In conclusion, we proposed a smart face mask incorporating a double-module sensor for multiple respiration pattern recognition. The smart face mask can be used to continuously monitor and recognize eight breath conditions, including nasal normal breath, nasal fast breath, nasal deep breath, cough, breath-holding, mouth normal breath and slow breath. The pressure sensing mode of combo sensor demonstrates high sensitivity that can measure small pressure generated by respiratory airflow, voice and arterial pulse. The temperature sensing mode shows noncontact detection of small temperature changes caused by mouth and nose breathing. The proposed smart face mask offers accurate discrimination between oral and nasal breathing in real time, which can be to prevent the development of mouth breathing syndrome. Also, the dual-mode sensing enables an unprecedented variety of breath status monitoring, allowing a more detailed analysis of the human body's physiology. The smart face mask has potential applications in monitoring the breathing patterns of patients with breathing-related diseases, such as COVID-19, pneumonia and so forth, which is beneficial for early warnings or diagnoses of disease.

## Methods

### Material

Polyacrylonitrile (PAN) was purchased from Macklin Sigma-Aldrich. The dimethylformamide (DMF) was purchased from Macklin. CNT (xfm04) was acquired from XFNAN. Poly (pyromellitic dianhydride-co-4,4′-oxydianiline), amic acid solution was acquired from Macklin, and it was used as precursors for PI film. PDMS (SYLGARD 184) was purchased from DOWSIL.

### Preparation of carbon nanofibers

Nanofiber precursors were fabricated via electrospinning. Firstly, the solution A was prepared by mixture of PAN and DMF with ratio 1:4 during magnetic stirring for 4 h. The solution B was prepared by adding 0.6 g CNT to 5 g DMF via ultrasonic dispersion over 2 h. Then, solutions A and B were mixed and magnetically stirred for 20 h to obtain the precursor solution. The precursor solution was loaded into a syringe and the feeding rate of the solution was (controlled) 2 mL h ^-1^. The distance between the tip of the needle and the covered collector was about 20 cm, and a high voltage (20 kV) was applied. The electrospun fibers were directly collected on a metal-foil-covered metallic rotating roller, typically performed over 2 h. Finally, the nanofibers were carbonized in a tube furnace at 900 °C in nitrogen for 2 h, and the temperature was increased with a rate of 20 °C min^-1^.

### Sensor fabrication

First, the pressure sensing mode was done by assembling the 3D buckling carbon nanofiber mats on top of a pair of interdigitated Au electrodes, and encapsulating with a 1.0 mil acrylic PSA adhesive, followed by covering with a 1.4 m-thick PEN film. The interdigitated Au electrodes (Au thickness = 100 nm; electrode width = 200 m; interval = 100 m; active pressure-sensitive area = 3 mm × 3 mm) were patterned on a 2.3 m-thick PI-coated Si-wafer using photolithography followed by magnetron sputtering. An anisotropic conductive wire was bonded to the interdigitated electrodes to connect them with standard DuPont pins. Second, the temperature sensing mode was prepared on the back side of the PI film of pressure mode. Two ends of the longitudinal carbon nanofibers were fixed by conductive silver paste and connected by copper wires with the external circuit. Then, the carbon nanofibers were covered by a PDMS precursor mixture (Sylgard 184, Dow Corning Corporation, prepolymer to crosslinker ratio varies from to 5:1) for 5 min to let the PDMS fully infiltrate the carbon nanofibers mats. The excess PDMS were then removed by spin-coating machine.

### Characterization of the material

The morphology of the carbon nanofibers was determined using field-emission scanning electron microscopy (Zeiss/Bruker Gemini500) and transmission electron microscopy (TEM, JEM-2100, JEOL). Raman spectroscopy was performed with a LabRAM HR 800 UV laser micro-Raman spectroscope (HORIBA Jobin Yvon, France), with a laser excitation wavelength of 532 nm. The Brunauer–Emmett–Teller (BET) surface analysis was performed with a surface area porosity analyzer (BSD-66).

### Device characterization platform

The pressure-sensing test was performed with an experimental setup consisting of a high-accuracy load cell (LSB200, USB200, FUTEK), a high-frequency piezoelectric actuator (NAP100, Newport), and a high-speed Keithley source-meter (Model 2636B). During the pressure-sensing test, the sensor was placed between the actuator and the load cell. The actuator was controlled to generate movement that can create pressure on the sensor, and the force was detected by the load cell. Temperature infrared images were captured using an infrared camera (Fluke TiX640). These sensors were connected to an acquisition card (Smacq-USB-3200), and the acquired pressure and temperature signals were record simultaneously.

### Ethics approval and consent to participate

This research was approved by the Ethics Committee of Sun Yat-sen University. All the experiments that include mask breathing and human skin temperature detection in this study were performed in accordance with guidelines and regulations. No other human subjects were involved in our experiments or manuscript. All participants signed an informed consent form prior to data collection.

## Supplementary Information

Supplementary Information 1.

Supplementary Video 1.

Supplementary Video 2.

Supplementary Video 3.

## Data Availability

The datasets used and/or analysed during the current study available from the corresponding author on request.
