# Supplementary material for: Flexible pressure and temperature dual-mode sensor based on buckling carbon nanofibers for respiration pattern recognition
Source: Sci Rep. 2022 Oct 19;12:17434. doi: 10.1038/s41598-022-21572-y (PMC9579593; doi:10.1038/s41598-022-21572-y)
Supplement: Supplementary file 1 — Supplementary Information 1. [file 41598_2022_21572_MOESM1_ESM.docx]

Supporting Information

**Flexible pressure and temperature dual-mode sensor based on buckling carbon nanofibers for respiration pattern recognition**

*Zhoujun Pang, Ningqi Luo, Yu Zhao, Dihu Chen,* Min Chen**


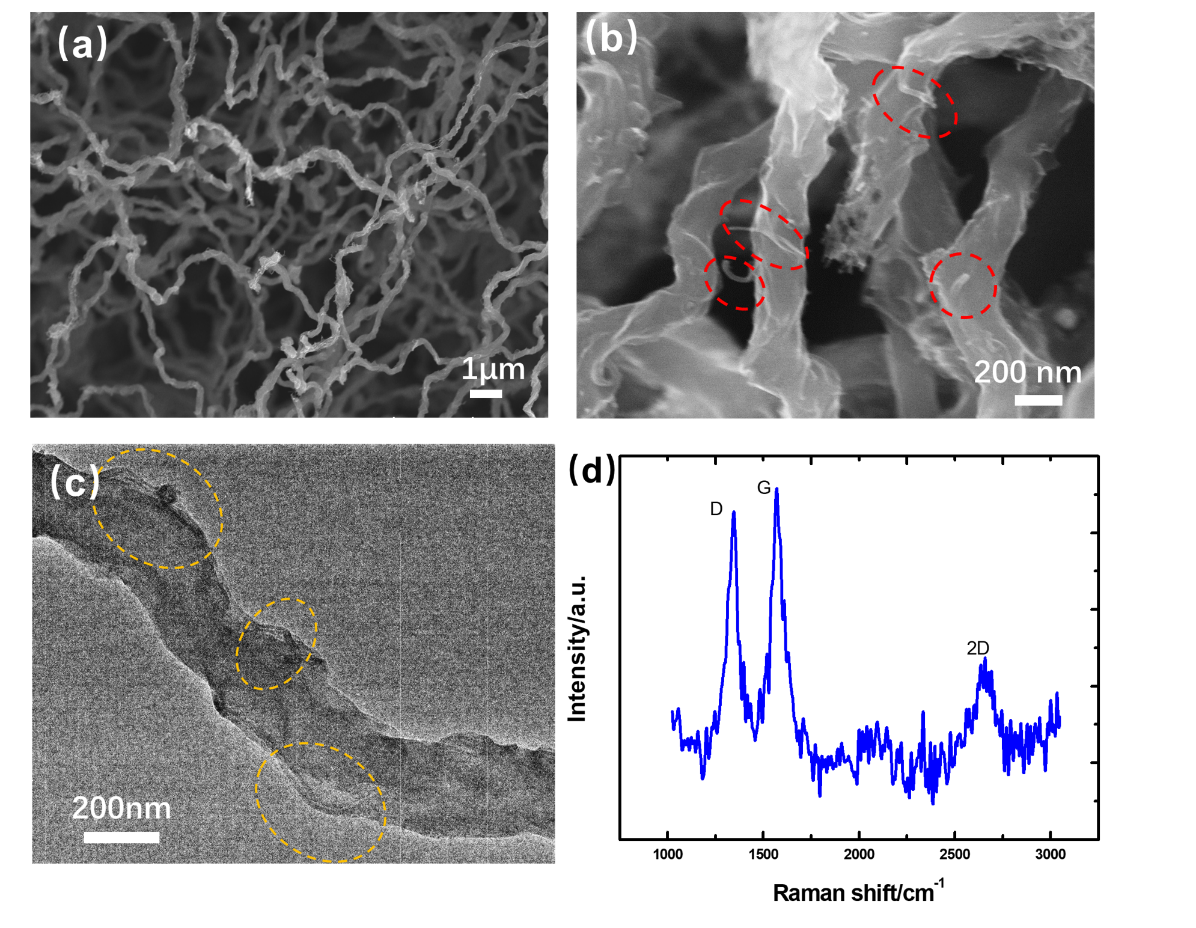


**Figure S1.** a) SEM image and b) High-resolution SEM image of CNF, c) TEM image and Raman spectra d) of CNF.


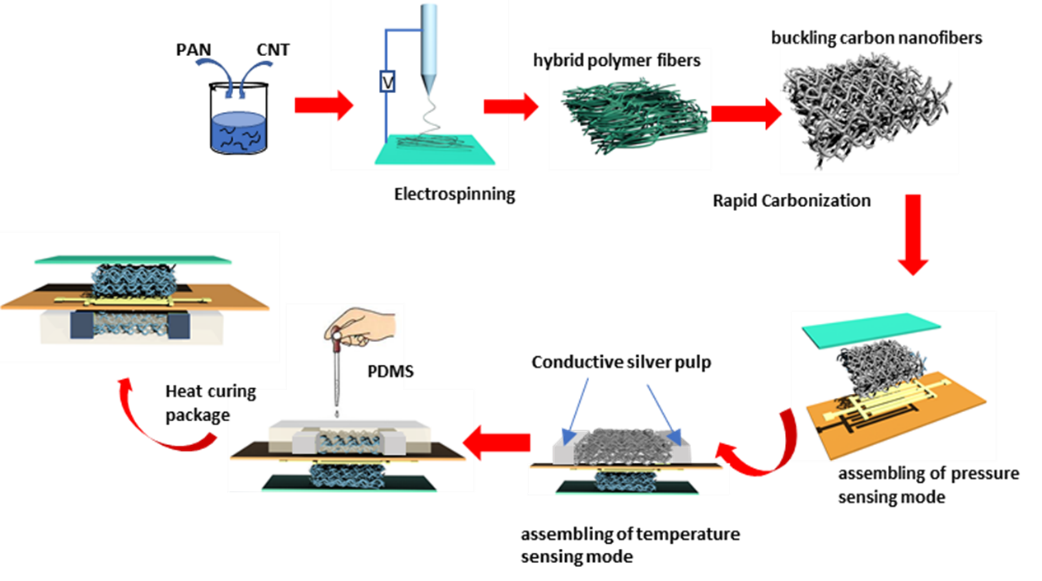


**Figure S2.** Schematic diagram of the preparation process of a dual-mode sensor


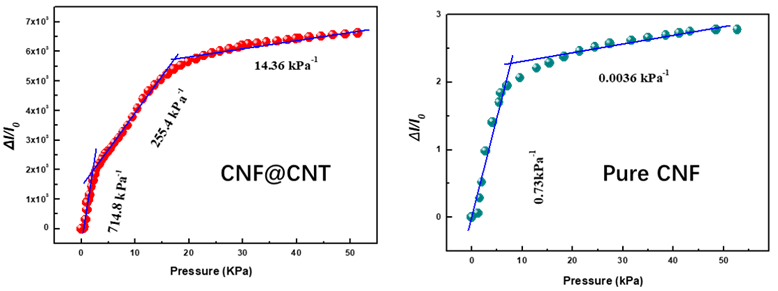


**Figure S3.** Comparison of sensitivity curves of different sensors.


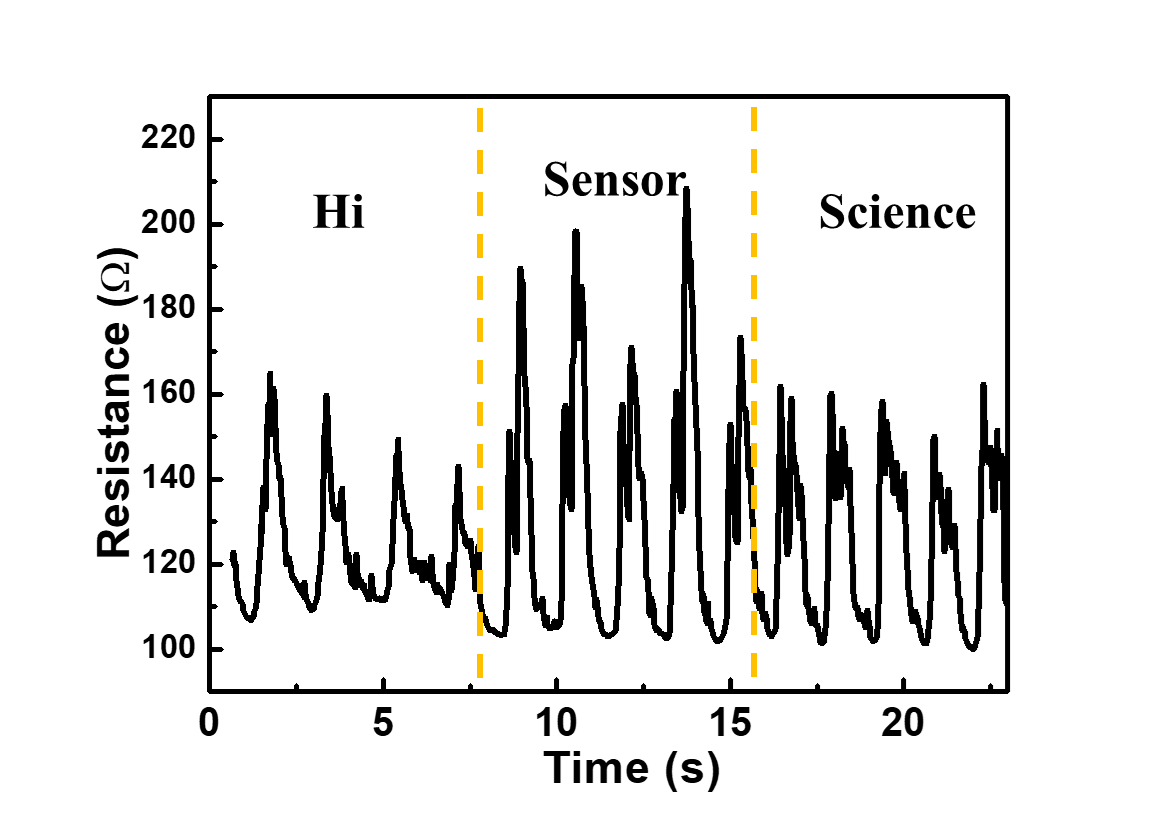


**Figure S4.** Responses of pressure sensor to words with different number of syllables.


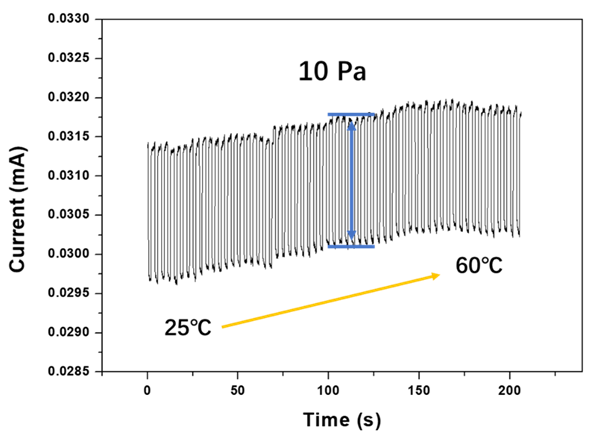


**Figure S5.** The pressure response under the change of temperature.


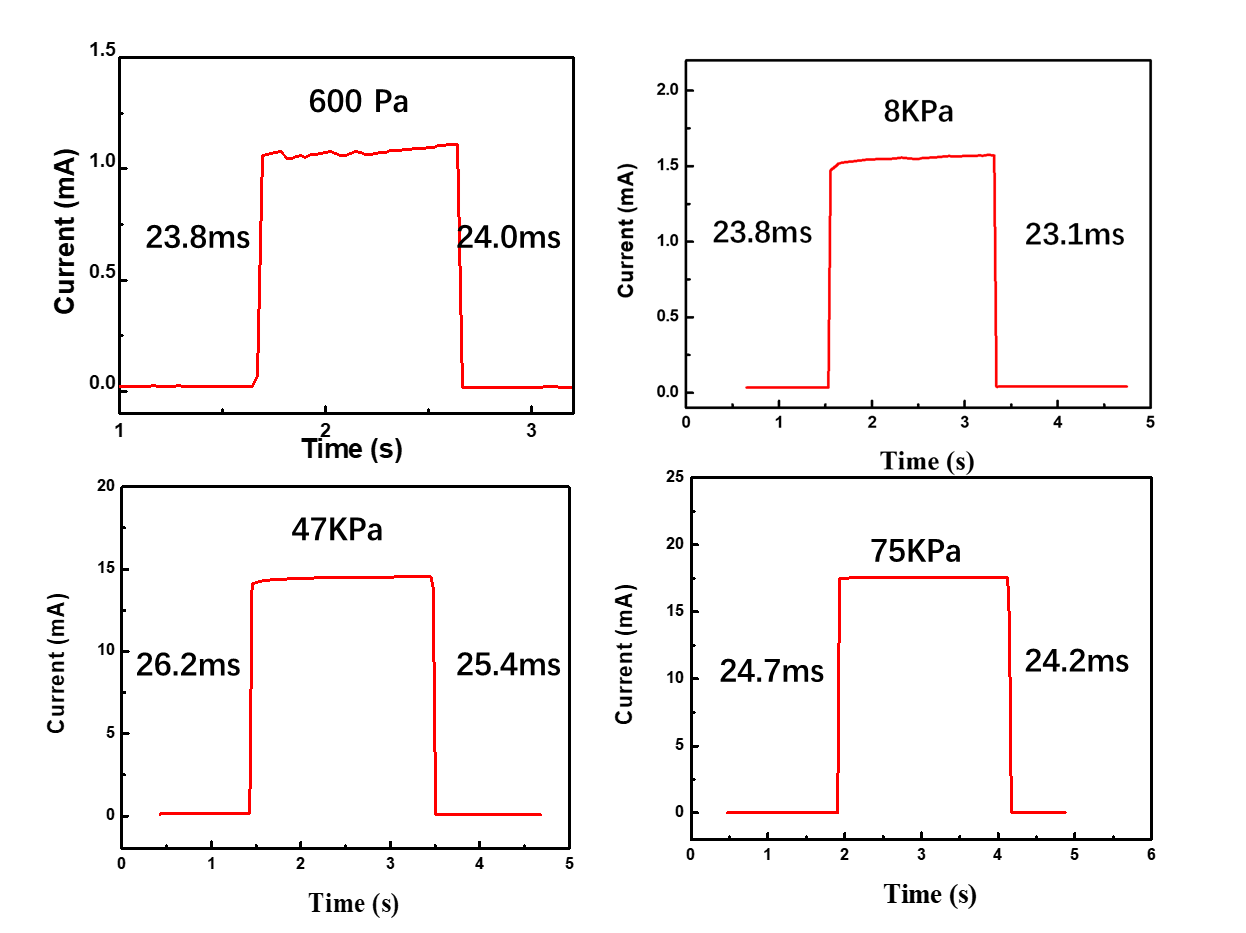
 **Figure S****6.** The response- and recovery- dynamic response of the sensor under 600 Pa, 8 kPa, 47 kPa and 75 kPa.


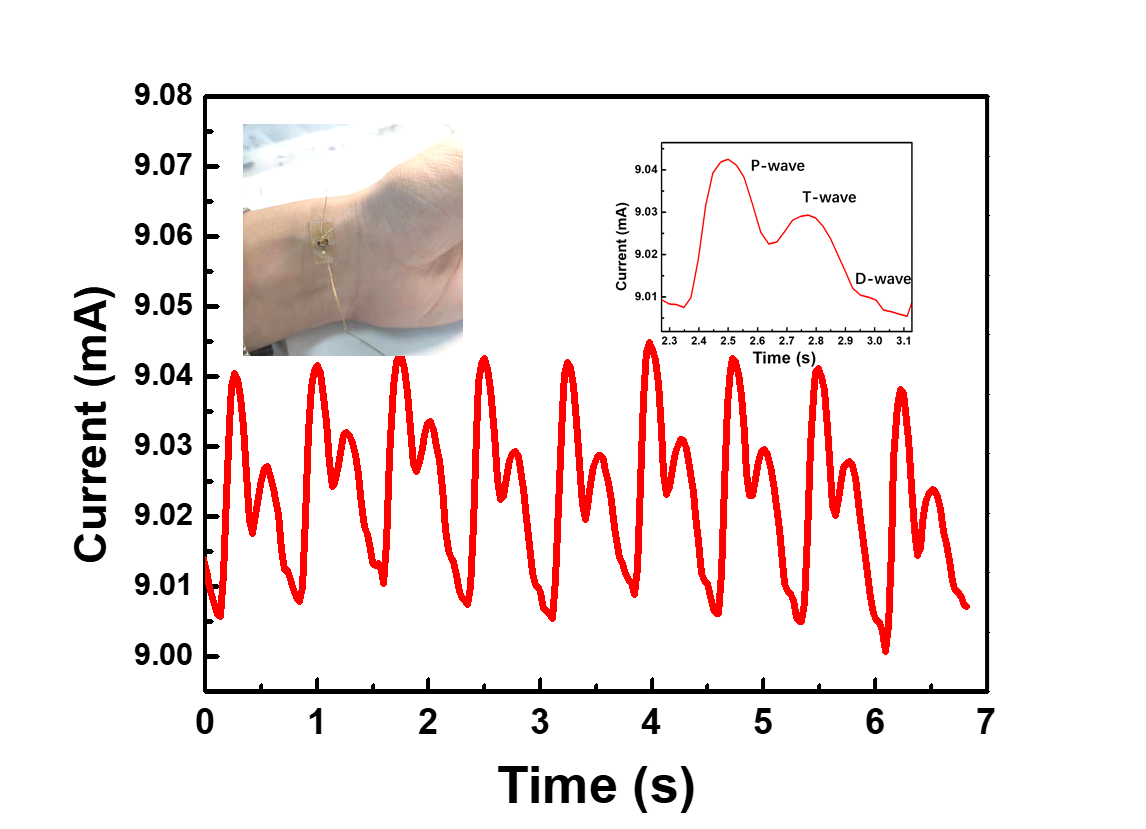


**Figure S7.** Flexible sensors is attached to the wrist for recording the arterial pulse signal.


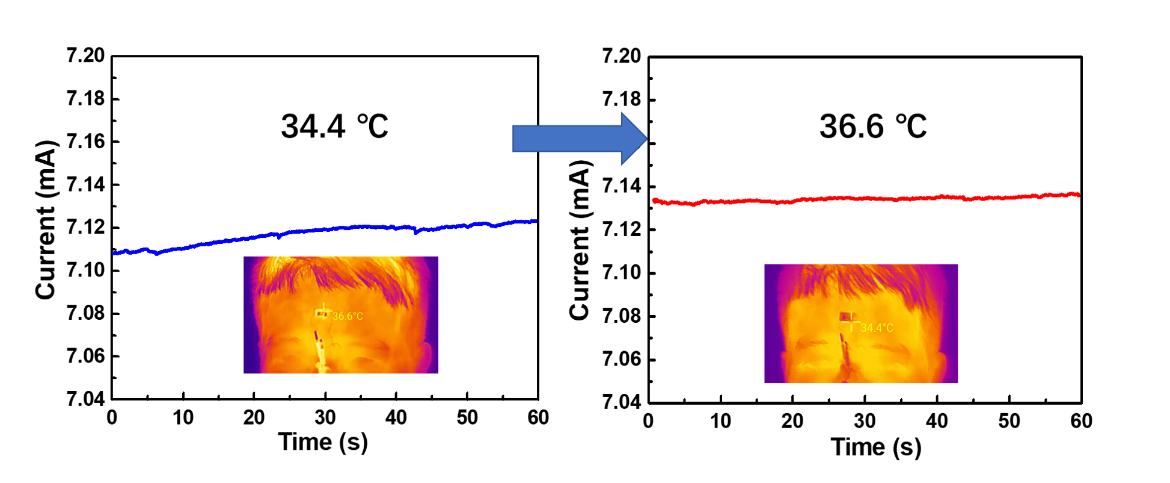
**Figure S8.** Current changes during the temperature rising and falling in an “artificial fever.” Insert image: IR thermal images of human forehead attached with our sensor.


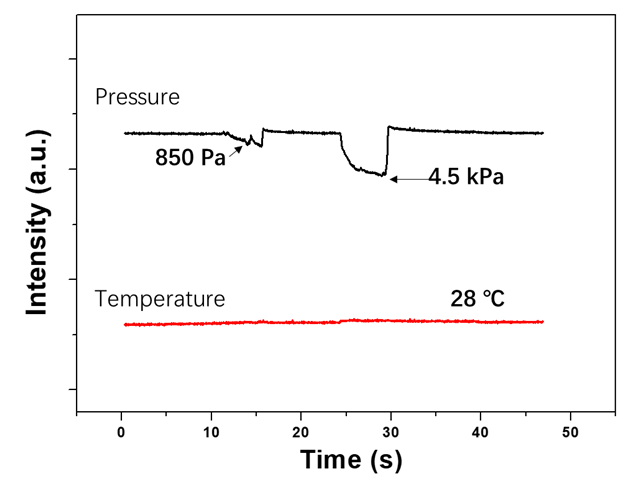


**Figure S9** Dual-mode sensor in response to sudden applied pressure.


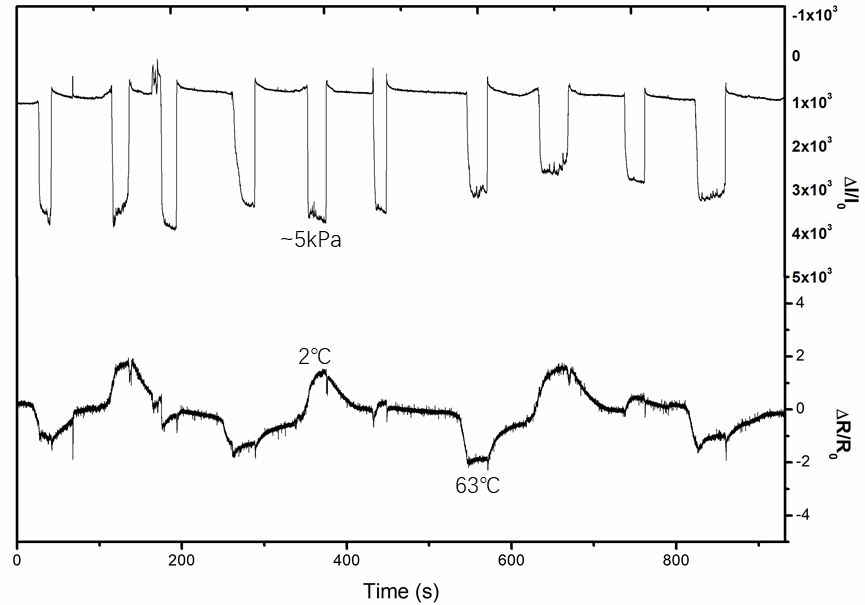


**Figure S10.** The repeated test of detecting temperature and pressure stimuli of objects at different temperatures.


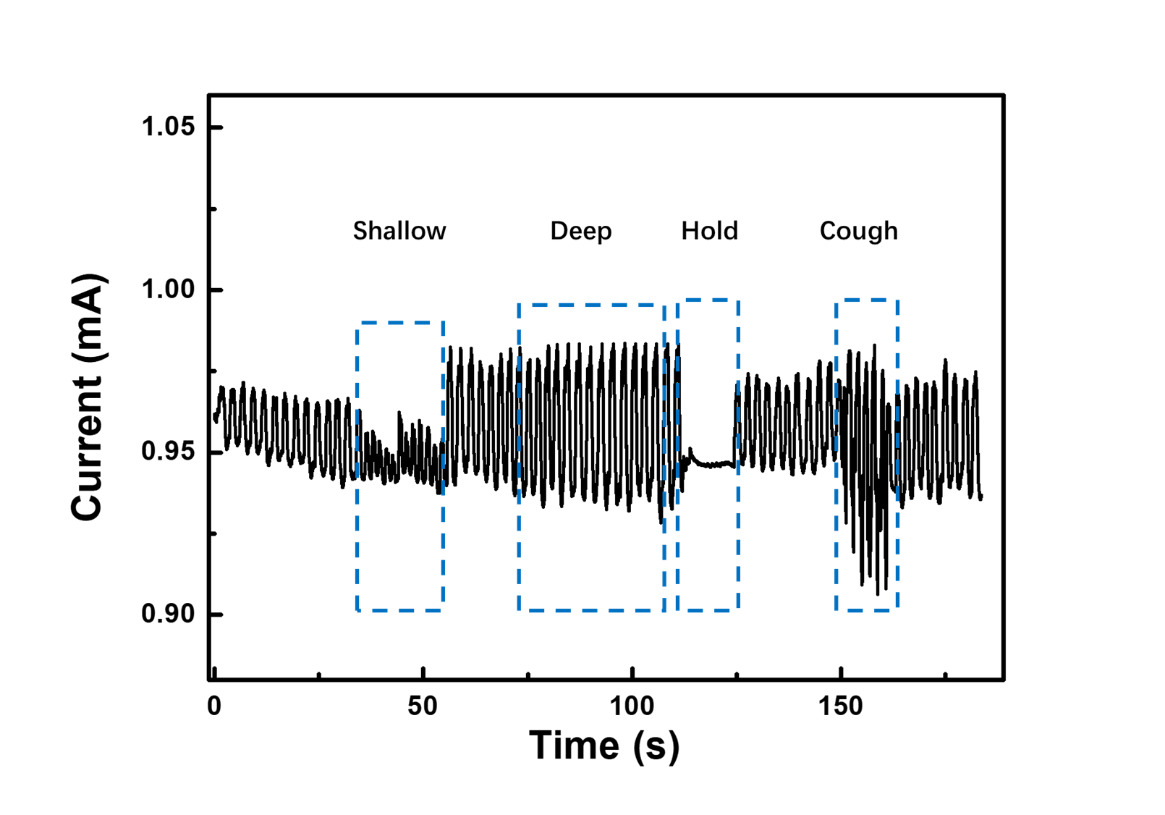


**Figure S11.** Continuous pressure signal test for multiple breathing patterns


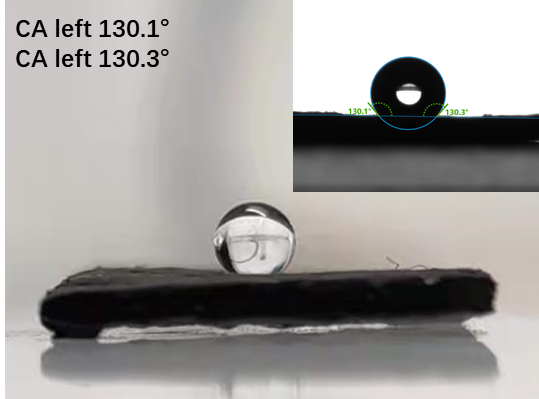


**Figure S12** Optical photograph of a water droplet on the surface of CNF. The inset shows the contact angle measurement of CNF, exhibiting high hydrophobicity with a contact angle of 130.

**Figure S13** Long-term breath monitoring in pressure sensing mode.


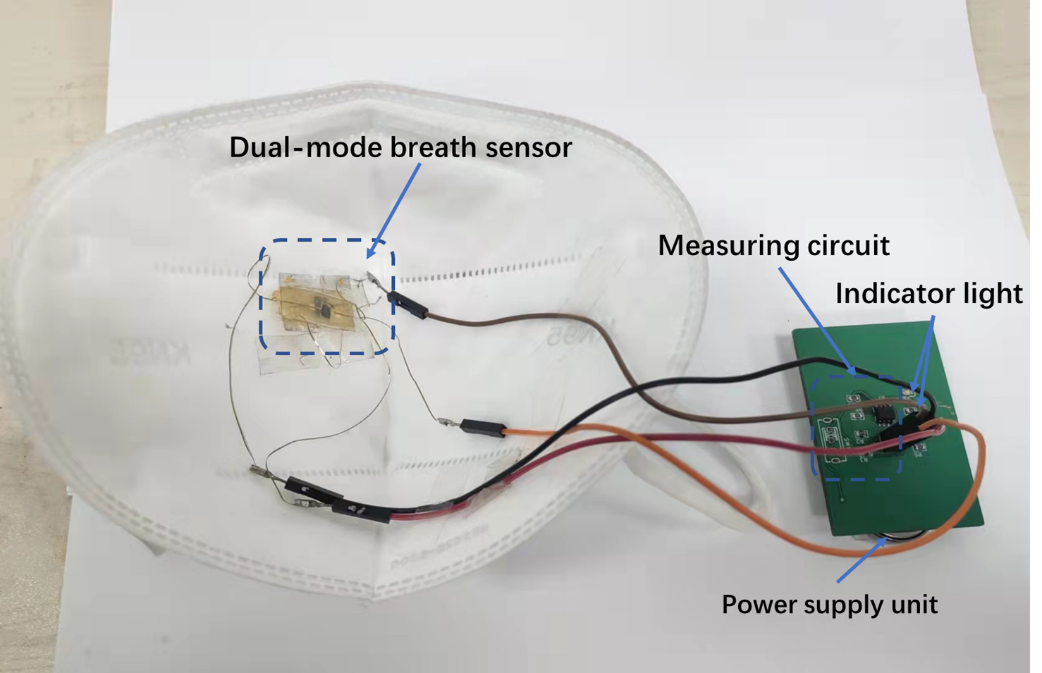


**Figure S14.** Photo illustrating of the smart mask for real-time recognition between mouth breath and nasal breath.


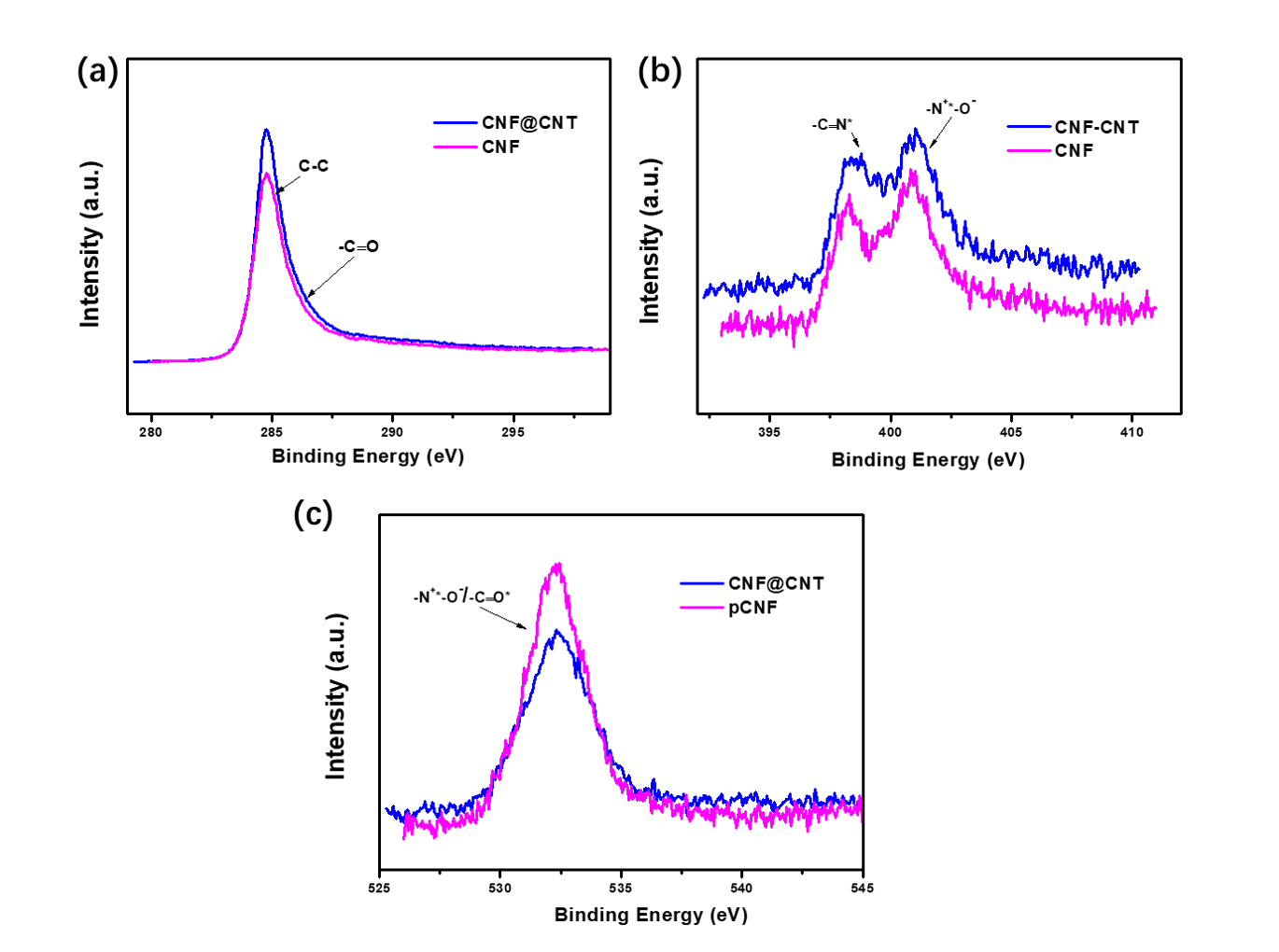


**Figure S15** XPS spectra of the CNF decorated with (CNF@CNT) and without (CNF) after the annealing at 900 °C.(a) C1s, (b) N1s, (c) O1s.

The XPS of CNF sample with and without the decoration of CNT after the annealing at 900 °C was measured. As shown in Figure S15, despite a slight change of peak intensity, the adding of CNT does not introduce any new peaks of functional groups in the C1s, N1s, and O1s spectra. This result suggests that the excellent sensing performance of the sensor originates from reasons other than the functional groups introduced by CNT.


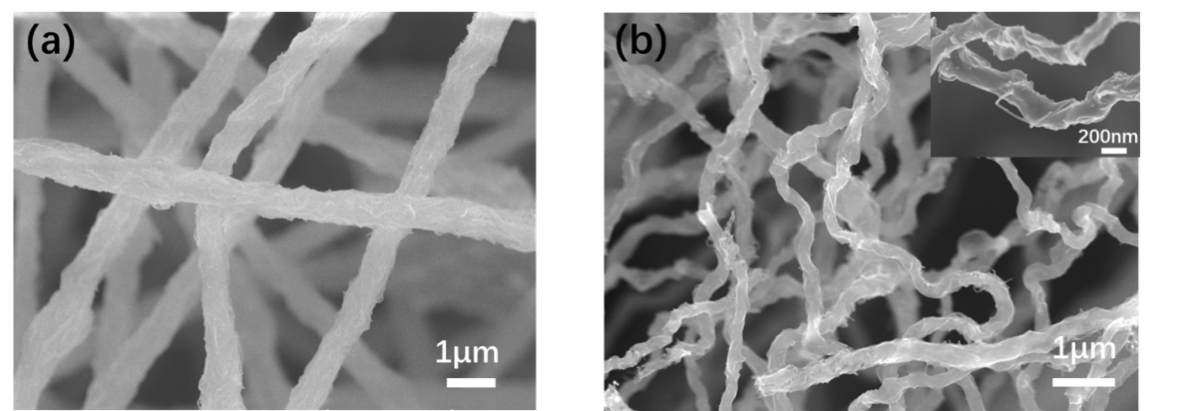


**Figure S16** SEM image of CNF before (a) and after (b) rapid annealing.

The SEM images of CNF sample before and after a rapid annealing of 900 °C are shown in Figure S16. The nanofiber becomes thinner and more curl after the annealing. Also, the protruding carbon nanotubes seem to be more obvious after annealing.


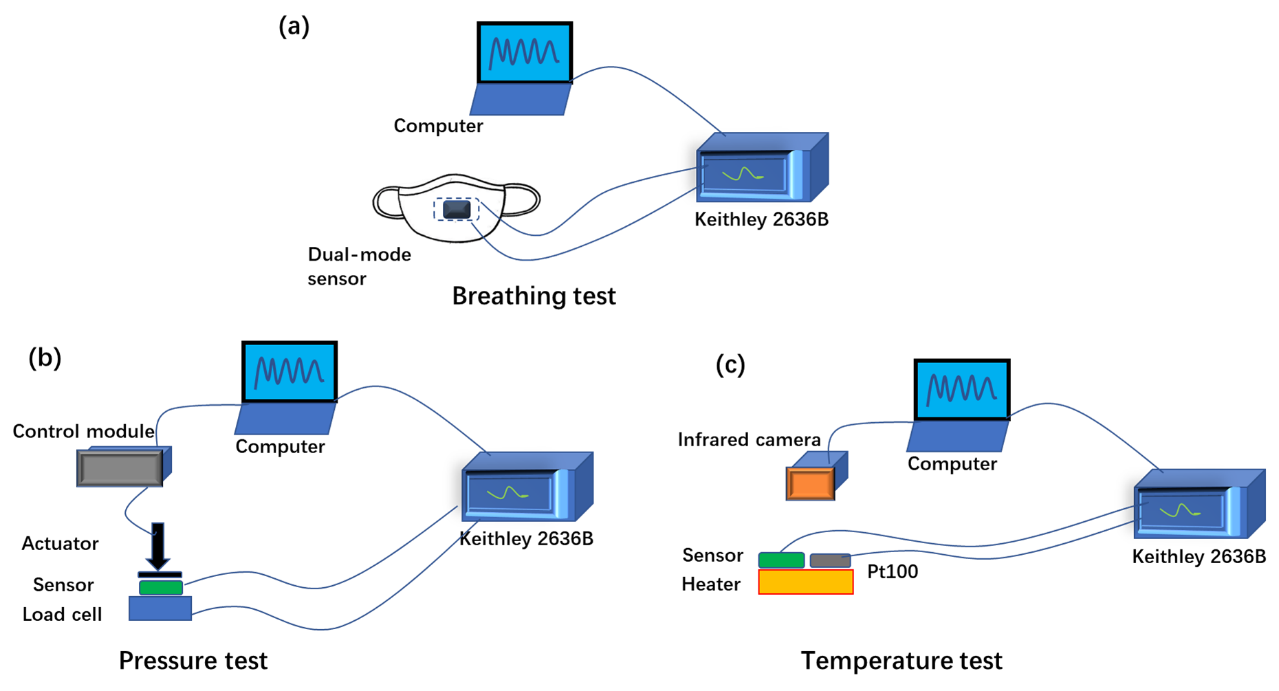


**Figure S17** The schematic of the experimental setup for (a) breathing test, (b)pressure test and (c) temperature test.


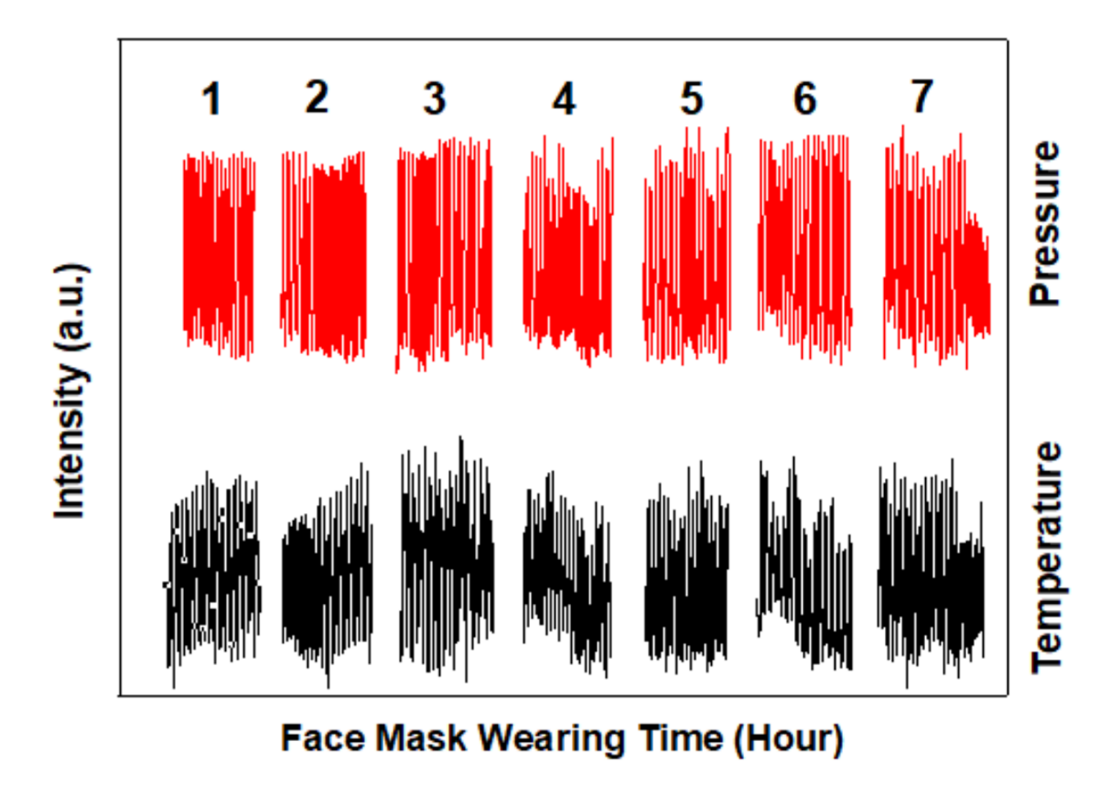


**Figure S18** The output stability test measured for 7 h.

**Figure S19** The pressure response at 0 °C, -10 °C and -20 °C.


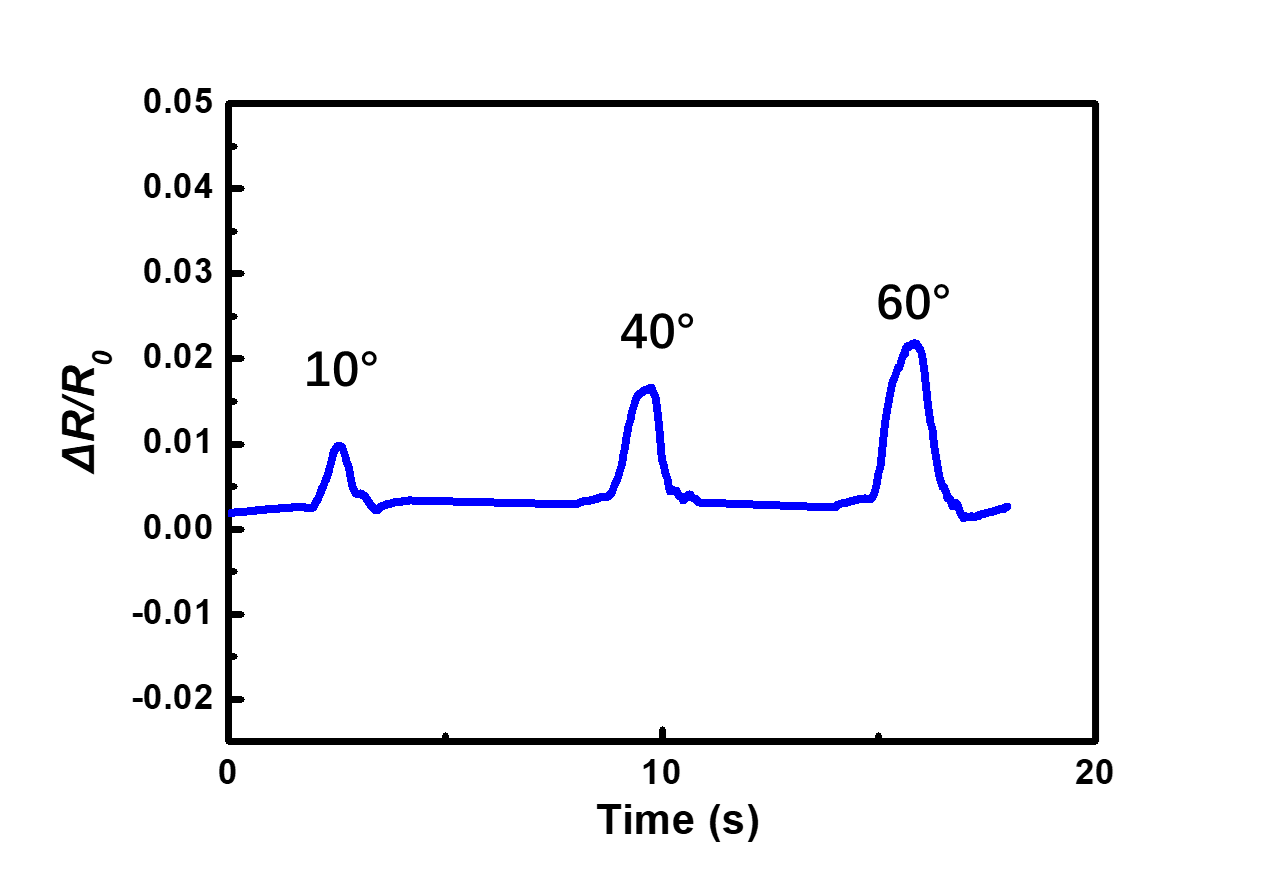


**Figure S20** The resistance variation of the sensor

under bending angles of ~15°, ~30° and ~60°.


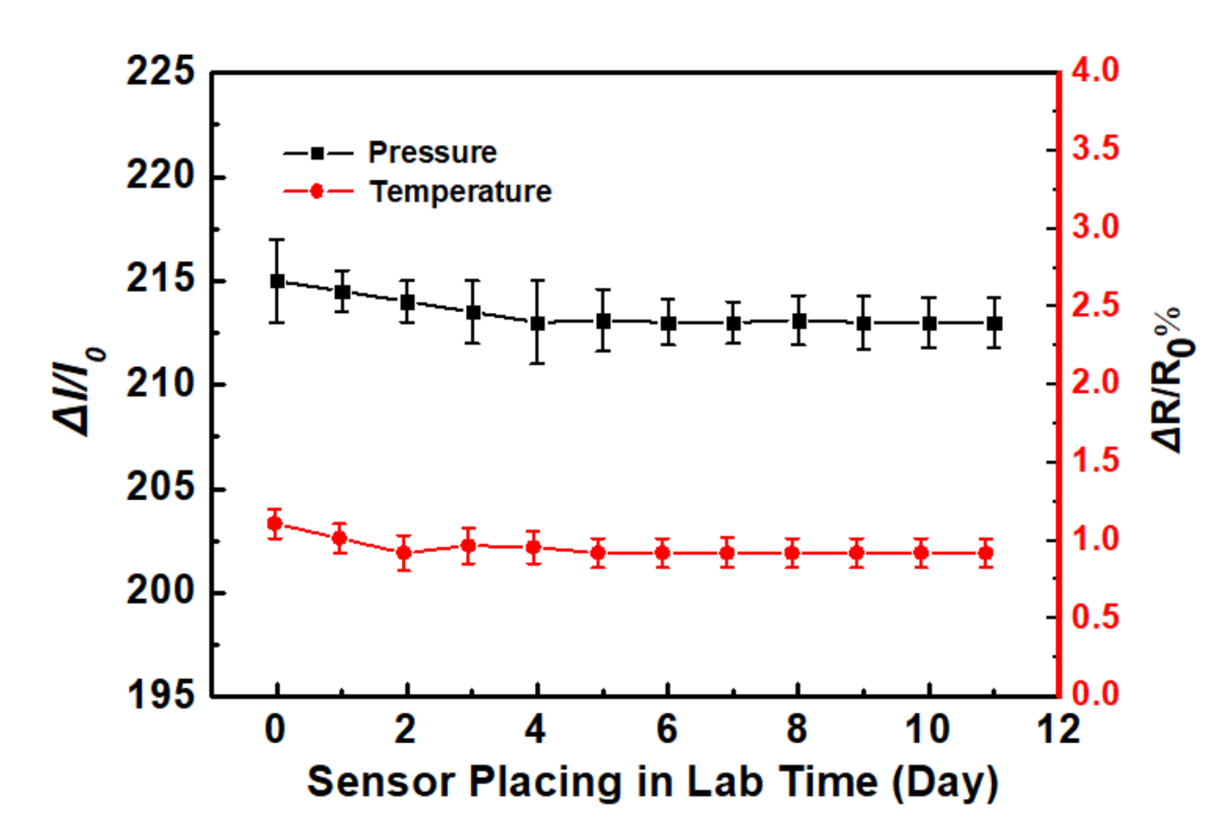


**Figure S21** The long-term output stability of the sensor.
